# Evaluation of Cortical Bone Formation on Mandibular Condyle in Asymptomatic Adolescents and Young Adults Using Cone-Beam Computed Tomography

**DOI:** 10.3390/life12122032

**Published:** 2022-12-05

**Authors:** Yo-Seob Seo, Hyun-Jeong Park, Sun-Kyoung Yu, Seo-Rin Jeong, Ji-Won Ryu

**Affiliations:** 1 Department of Oral and Maxillofacial Radiology, School of Dentistry, Gwangju 61452, Republic of Korea; 2 Department of Oral Medicine, School of Dentistry, Gwangju 61452, Republic of Korea; 3 Department of Oral Anatomy, School of Dentistry, Gwangju 61452, Republic of Korea; 4 Department of Orthodontics, School of Dentistry, Gwangju 61452, Republic of Korea

**Keywords:** adolescent, cortical bone, CBCT, mandibular condyle, orthodontic treatment, temporomandibular joint, temporomandibular disorder

## Abstract

The aim of this study was to evaluate cortical bone formation on the mandibular condyle using cone-beam computed tomography (CBCT) in asymptomatic adolescents and young adults and to evaluate the relationship between age and sex. CBCT images that can evaluate the shape of the mandibular condyle were selected from asymptomatic patients aged 13–25. The degree of cortication on the mandibular condyle (CMC) was evaluated using CBCT images reconstructed in the axial, sagittal, and coronal planes. CBCT data of 829 patients (413 males, 416 females) were selected and then the left and right images of all patients were acquired; consequently, a total of 1658 temporomandibular joint-related images were evaluated in this study. The degree of CMC was correlated with age in men and women (*p* < 0.05). The frequency of CMC 0 disappeared in woman aged 20 years and in men aged 21 years. Cortical bone formation of the mandibular condyle was completed at age 22 years in women and 24 years in men. The degrees of cortical bone formation of the mandibular condyle between men and women showed significant differences between the ages of 15–19 and 22 years. This difference can be interpreted as a different mandible growth period between the sexes.

## 1. Introduction

Temporomandibular disorder (TMD) is a collective term that describes a group of disorders that affect the masticatory system: temporomandibular joint (TMJ) and masticatory muscles [1]. It has been known that the prevalence of TMD for age follows an inverted U-shaped curve [2]. Moreover, research and clinical guidelines for TMD have mainly targeted the adult population. However, it has been reported that orofacial pain is the main cause of health counseling and dental appointments among adolescents [3]. In addition, patients with TMD in late adolescence reported higher orofacial pain intensity than early adolescence patients, with a more significant impact on daily activities [4].

Unlike TMDs in adults, the growth and development of related tissues can affect the timing of diagnosis and treatment of TMDs in children and adolescents. The body of mandible and ramus begin to grow in childhood (5–6 years), and the width of the mandible is determined in early adolescence [5]. As the length of the mandible increases, the secondary cartilage of the mandibular condyle acts as the secondary growth center of the mandibular condyle and the skull [5,6]. Therefore, if the normal growth pattern is altered and these abnormalities are not appropriately treated, the mandible, the maxilla, and the midface can be deformed [7]. These acquired functional deformities of the TMJ can be broadly classified as undergrowth/destructive or overgrowth patterns [8]. In general, the longer the disease duration that can cause abnormalities in TMJ, the higher the risk of orofacial deformity, and the possibility of physical limitations for tasks such as mastication and conversation may increase, so early diagnosis of the disease and its treatment are mandatory [9,10]. Orthodontic treatment and orthognathic surgery may be considered to treat orofacial deformities. The timing of such treatment depends on the severity of the mandibular condyle and the activity of the disease [8].

Among the imaging techniques related to the TMJ, cone-beam computed tomography (CBCT) is known to be easy to use to evaluate osseous tissue [11]. CBCT is known as the gold standard imaging technique for hard tissue evaluation in patients with degenerative joint disease [12] and can be easily performed in dental practice. When evaluating abnormalities of the mandibular condyle in children or adolescents, the criteria for degenerative osteoarthritis are mainly used, which assess the continuity of the cortical bone above the mandibular condyle and the shape of the condyle [13]. However, the mandible is the last bone to mature in the body [8], and the cortical bone from the fibrous cartilage of the mandibular condyle completes its formation in the early 20s [14]. The lack of knowledge about the growth of the mandible in children and adolescents is a significant barrier to diagnosing and treating diseases related to the mandibular condyle: TMD and malocclusion. Therefore, this study aims to evaluate cortical bone formation on the mandibular condyle using CBCT in asymptomatic adolescents and young adults and to evaluate the relationship between age and sex.

## 2. Materials and Methods

### 2.1. Subjects

In this study, among the CBCT images of patients aged 13–25 years at the time of imaging at Chosun University Dental Hospital, images showing the shape of the mandibular condyle were selected.

Exclusion criteria consisted of imaging to evaluate TMD, images showing destructive change on the mandibular condyle surface, head and neck diseases including the mandibular condyle, and chronic or systemic diseases that could affect the growth and development of the jaw. Additionally, after reviewing the electronic medical records, those with a trauma history including the face and jaws, orthodontic treatment history, and symptoms and/or treatment history of TMD were also excluded from this study.

Accordingly, CBCT data of 829 patients (413 males, 416 females) were selected and then left and right images of all patients were acquired; thus, a total of 1658 TMJ-related images were evaluated in this study.

This retrospective study was approved by the Institutional Review Committee of the Chosun University Dental Hospital (CUDHIRB-1901 011 R02).

### 2.2. Image Reconstruction

**Figure 1:**
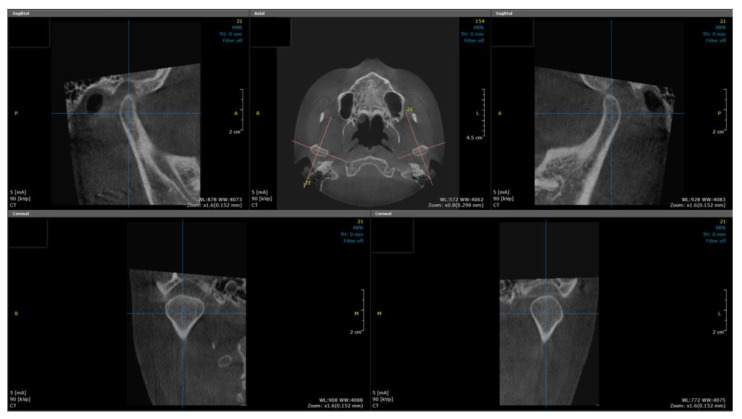
Sagittal and coronal reconstruction of a cone beam computed tomography.

The CBCT imaging machines we used were CS9300 (Carestream Health Inc., Rochester, NY, USA) and Planmeca Viso G7 (Planmeca, Helsinki, Finland), under 80–120 kVp, 5–11 mA, and voxel size 0.25–0.3 mm. The software used to reconstruct and analyze CBCT images was OnDemand3D (Cybermed Co., Seoul, Republic of Korea). To reconstruct the image with the left and right mandibular condyles located at symmetrical positions in the axial and coronal planes, we adjusted the axial plane of the image to the occlusal plane of the mandible. Then, the coronal plane of the condyle was reconstructed along the horizontal axis of the mandibular condyle, and the sagittal plane of the condyle was obtained to be perpendicular to the coronal plane of the mandibular condyle (Figure 1). Each plane was reconstructed at 1.0 mm slice intervals.

### 2.3. Evaluation of Cortical Bone Formation on the Mandibular Condyle

**Figure 2:**
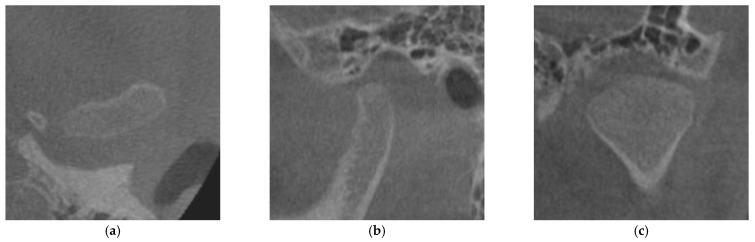
Image of the mandibular condyle with sagittal, coronal, and axial reconstructions evaluated as CMC 0: (**a**) axial reconstruction; (**b**) sagittal reconstruction; (**c**) coronal reconstruction. CMC 0, no cortical bone exists on the articular surface of the mandibular condyle.

**Figure 3:**
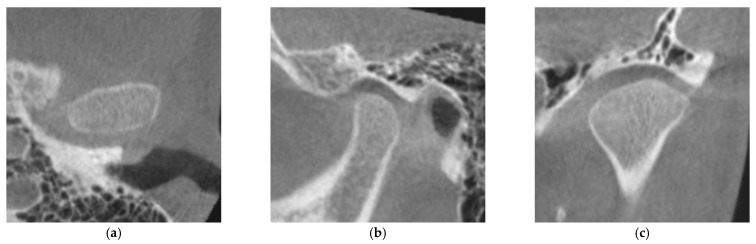
Image of the mandibular condyle with sagittal, coronal, and axial reconstructions evaluated as CMC 1: (**a**) axial reconstruction; (**b**) sagittal reconstruction; (**c**) coronal reconstruction. CMC 1, cortical bone is partially present in the articular surface of the mandibular condyle.

**Figure 4:**
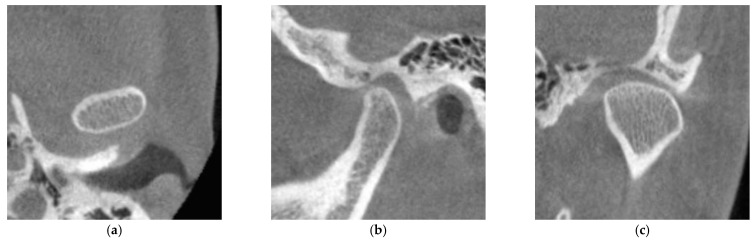
Image of the mandibular condyle with sagittal, coronal, and axial reconstructions evaluated as CMC 2: (**a**) axial reconstruction; (**b**) sagittal reconstruction; (**c**) coronal reconstruction. CMC 2, cortical bone completely covers the articular surface of the mandibular condyle.

The degree of cortication on the mandibular condyle (CMC) was evaluated on the reconstructed images. We evaluated the degree of CMC according to the classification of Lei [15]. Briefly, CMC is divided into three groups: CMC 0, no cortical bone exists on the articular surface of the mandibular condyle (Figure 2); CMC 1, cortical bone is partially present in the articular surface of the mandibular condyle (Figure 3); CMC 2, cortical bone completely covers the articular surface of the mandibular condyle (Figure 4).

Two experts (YSS in oral and maxillofacial radiology and JWR in oral medicine) with more than ten years of clinical experience evaluated CMC. As a preliminary task before entering the final assessment, the process of randomly selecting 30 subjects for evaluation was performed to improve inter-examiner agreement and increase the reliability of this evaluation. If there was disagreement between examiners in the first evaluation, a discussion was conducted to reduce the evaluation gap. Subsequently, each examiner independently performed the evaluation process twice. During the evaluation process, variables (age, gender) that could affect the study results were blinded.

### 2.4. Statistical Analysis

Cohen’s Kappa coefficient (k) was used to calculate inter- and intra-examiner agreement of CMC evaluation [16]. The concordance of left and right CMCs was also analyzed using Cohen’s Kappa coefficient. The number of each variable in this study is more than 30. Therefore, it is assumed to have normality according to the central limit theorem.

The variables used in this study belong to a categorical scale, so descriptive statistics including frequency analysis was performed to evaluate the difference in CMC according to age and gender. Spearman correlation analysis was performed to analyze the correlation between CMC and age. Differences between men and women within the same age were evaluated using the chi-square test.

All statistical analyses were performed using IBM SPSS Statistics for Windows, version 26.0 (IBM Co., Armonk, NY, USA). A *p*-value < 0.05 was regarded as statistically significant.

## 3. Results

CBCT data of 829 patients (413 males, 416 females) were chosen, and each CBCT data included both sides of the mandibular condyle. The concordance of left and right CMCs was also analyzed using Cohen’s Kappa coefficient. The Cohen-Kappa coefficient between the left and right CMC values was k = 0.589, confirming no difference between the left and right measured values. Therefore, we integrated the left and the right-side evaluation values into one evaluation value without separating them. Thus, the subjects of this study were 1658 images of TMJs in total. The distribution of the number of subjects by age and sex is described in Table 1.

### 3.1. Inter-Examiner/Intra-Examiner Reliability

The Cohen-Kappa value was 0.932, indicating a very high agreement between the examiners in evaluating the degree of CMC. The intra-examiner agreement of the second examiner was k = 0.984, indicating a very high level of reliability. Therefore, we conducted this study by adopting the second evaluation value of the second examiner.

### 3.2. Frequency Distribution of the Degree of CMC according to Age and Sex

Frequency analysis of the degree of CMC was expressed as the observed number and percentage according to age and sex (Table 1).

Most subjects (80.4% of boys and 65.5% of girls) at the age of 13 were evaluated as CMC 0, and CMC 1 was also observed. However, there was no case corresponding to CMC 2. CMC 2 began to be recorded at the age of 14 in women and 17 in men.

The cortical bone formation on the mandibular condyle progresses slowly with age. Using the Spearman correlation coefficient, the degree of CMC was correlated with age in both men and women (*p* < 0.05). In women, the frequency of CMC 0 disappeared from the age of 20. In addition, the percentage of CMC 2 increased remarkably to 73.0% between the ages of 21 and 22, and a similar frequency was observed at later ages. In men, the frequency of CMC 0 disappeared from age 21. The percentage of CMC 2 increased to 75.8% between the age of 23 and 24, and it showed a gradual increase until 25.

Chi-square correlation analysis showed a significant difference in the degree of CMC between sex at the same age, and the difference was between 15–19 and 22 years of age.

## 4. Discussion

This study evaluated the degree of CMC according to age and sex to investigate the relationship between these variables. We also tried to determine the age by which CMC was completed in male and female adolescents and young adults. The subjects in this study showed a uniform number of subjects for each age, both men and women.

Most condylar cartilage is replaced by bone at birth, but its superior portion remains involved in mandibular growth [5]. The fibrocartilage on the articular surface is transformed into the compact bone by the load generated by various dynamic mandibular movements [17]. The cortical bone transfers the load to the fibrocartilage layer of the condyle within physiological limits [6]. However, continuous overload, such as clenching, can cause a partial loss of cortical bone on the mandibular articular surface [9]. In addition, one of the criteria for degenerative changes in the TMJ is the density or continuity of the cortical bone of the articular surface [18]. Therefore, this study evaluated the degree of CMC.

Various imaging techniques can be used to study the shape and growth of the mandible [19,20] and we used CBCT to reconstruct and evaluate the cortical bone formation on the mandibular condyle. CBCT provides a lower dose compared to conventional computed tomography (CT) and provides high-resolution images of bone tissue [21]. Compared to conventional tomography, CT, micro-CT, and microscopy, CBCT is superior in recognizing minute changes in the mandibular condyle cortex [22]. Therefore, we performed this study using CBCT, which readily detects bone morphology of the mandibular condyle.

In this study, the degree of CMC was correlated with age in men and women. Recent studies on the correlation between condylar cortication and age have also been reported in legal medicine [14,21,23]. However, adolescent female patients with TMJ osteoarthritis showed delayed tooth development and craniofacial growth disorders without skeletal maturation alteration [24]. Therefore, age estimation using condylar cortication is not suitable for people with TMD or any disease that can affect TMJ.

Comparing CMC degrees at the same age showed a difference between men and women aged 15–19 years and 22 years (Table 1). To our knowledge, this study is the first to demonstrate a difference in condylar cortical bone formation between men and women at a specific age. This difference can be interpreted as a different mandible growth period between the sexes. As for the growth and development of the mandible, the width is determined first, and the mandibular length then increases [5]. The height of the lower jaw is completed around the age of 20 [25]. In this study, complete cortical bone formation (CMC 2) began to appear at the age of 14 years in women and 17 years in men. The CMC 2 appearance time gap could be a factor that showed a significant difference between men and women aged 15–19 years. In women, the frequency percent of CMC 2 increased significantly to 73.0% between the ages of 21 and 22, and a similar frequency was observed in later age groups. Therefore, we inferred that complete cortical bone formation on the mandibular condyle occurs at the age of 22 years in women. These results might cause a difference in CMC between men and women aged 22 years. In men, the frequency percent of CMC 2 increased significantly to 75.8% between the ages of 23 and 24 years, with a gradual increase until 25 years. However, there was no difference in the degree of CMC between men and women at 24, which can be explained by the fact that women had already completed cortical bone formation on the condyle. In several studies evaluating mandibular condyle cortication, complete cortical bone formation timing showed varying results [21,23,26]. Different results for each study may be due to differences in race, the number of subjects, and the evaluation criteria. A previous study with the same evaluation criteria [15] showed similar trends to this study, but the results of this study were slightly delayed.

For subjects aged 13 years, most samples (80.4% of boys and 65.5% of girls) were evaluated as CMC 0. This result is similar to a previous study, although that study reported cortical bone formation in boys started at the age of 13–14 years [15]. In contrast, in this study, cortical bone formation was already in progress in 13-year-old boys. Different results with the same criteria may be due to racial differences, although differences in the timing of the study may also be an influencing factor. Therefore, it will be necessary to conduct follow-up studies with subjects of a broader age range after a certain period.

This study has implications for clinicians treating patients with TMD or malocclusion among adolescents and young adults. In this study, the degree of CMC correlated with age, and CMC 0 and CMC 2 were not observed in any specific age group. Thus, if women less than 14 years or men less than 17 years show complete cortical formation on mandibular condyles, and if women aged more than 20 years or men aged more than 21 years have no cortical formation, their mandibular condyles may have interrupted growth patterns. Clinicians should try to find out what caused growth alterations in the mandibular condyle.

Complete cortical bone formation of the mandibular condyle was reached at 22 years in women and 24 years in men. Excessive overload or trauma to the articular surfaces of the growing condyle can lead to dysfunctional remodeling of the condyle, leading to osteoarthritis. Therefore, clinicians need to be careful not to perform dental procedures that can cause extensive and abrupt changes in the masticatory system to patients in the growing state. Additional studies on the relationship between jaw deformity and growth of the mandible are needed to effectively treat and predict the prognosis of patients with jaw deformities that require significant changes in the masticatory system, such as in orthognathic surgery.

Finally, in this study, the level of CMC 2 was approximately 75%, even at the age of maximum maturity. The CMC 2 value was approximately 75% even at an age when the cortical bone of the mandibular condyle was fully formed. It should be considered that TMJ undergoes the process of forming cortical bone, which continues to remodel throughout life [13]. Therefore, when treating patients with TMD or malocclusion, clinicians should be careful not to diagnose degenerative osteoarthritis using only images without considering the clinical symptoms.

## 5. Conclusions

Cortical bone formation on the mandibular condyle was correlated with age in both men and women. The degree of cortical bone formation of the mandibular condyle between men and women showed a significant difference between the ages of 15–19 and 22. This difference can be interpreted as a different mandible growth period between sexes. Cortical bone formation of the mandibular condyle was completed at 22 in women and 24 in men. Understanding the mandibular condyle growth patterns will benefit clinicians diagnosing and treating patients with TMD or malocclusion in adolescence.

## Figures and Tables

**Table 1:** Frequency distribution of the degree of cortication on the mandibular condyle according to age and sex.

Men				Women				Chi-Square Test	
Age (*n*)	CMC 0	CMC 1	CMC 2	Age (*n*)	CMC 0	CMC 1	CMC 2	*p*-Value	χ^2^, df
13 (56)	45 (80.4)	11 (19.6)	0 (0)	13 (66)	38 (65.5)	20 (34.5)	0 (0)	0.075	3.17, 1
14 (66)	51 (77.3)	15 (22.7)	0 (0)	14 (66)	41 (62.1)	23 (34.8)	2 (3.0)	0.761	0.09, 1
15 (64)	48 (75.0)	16 (25.0)	0 (0)	15 (64)	35 (54.7)	27 (42.2)	2 (3.1)	0.033 *	6.85, 2
16 (62)	36 (58.1)	26 (41.9)	0 (0)	16 (64)	24 (37.5)	36 (56.3)	4 (6.3)	0.018 *	7.98, 2
17 (66)	34 (51.5)	30 (45.5)	2 (3.0)	17 (64)	18 (28.1)	37 (57.8)	9 (14.1)	0.006 *	10.08, 2
18 (66)	32 (48.5)	31 (47.0)	3 (4.5)	18 (64)	6 (9.4)	46 (71.9)	12 (18.8)	0.000 *	26.09, 2
19 (66)	7 (10.6)	50 (75.8)	9 (13.6)	19 (64)	1 (1.6)	43 (67.2)	20 (31.3)	0.010 *	9.17, 2
20 (62)	2 (3.2)	47 (75.8)	13 (21.0)	20 (62)	0 (0)	42 (67.7)	20 (32.3)	0.152	3.77, 2
21 (64)	0 (0)	41 (64.1)	23 (35.9)	21 (64)	0 (0)	31 (48.4)	33 (51.6)	0.075	3.18, 1
22 (66)	0 (0)	35 (53.0)	31 (47.0)	22 (63)	0 (0)	17 (27.0)	46 (73.0)	0.003 *	9.09, 1
23 (66)	0 (0)	23 (34.8)	43 (65.2)	23 (63)	0 (0)	15 (23.8)	48 (76.2)	0.169	1.89, 1
24 (66)	0 (0)	15 (24.2)	48 (75.8)	24 (64)	0 (0)	16 (25.0)	48 (75.0)	0.916	0.01, 1
25 (60)	0 (0)	13 (21.7)	47 (78.3)	25 (66)	0 (0)	16 (24.2)	50 (75.8)	0.732	0.12, 1

*n*, number; CMC 0, no cortical bone exists on the articular surface of the mandibular condyle; CMC 1, cortical bone is partially present in the articular surface of the mandibular condyle; CMC 2, cortical bone completely covers the articular surface of the mandibular condyle; χ^2^, χ^2^ value; df, degrees of freedom. Values are presented observed number (percentage). Percentages are rounded to one decimal place. * *p* < 0.05, chi-square test.

## Data Availability

Not applicable.
